# Modification of Commercial 3D Fused Deposition Modeling Printer for Extrusion Printing of Hydrogels

**DOI:** 10.3390/polym14245539

**Published:** 2022-12-17

**Authors:** Semyon I. Koltsov, Tatiana G. Statsenko, Sofia M. Morozova

**Affiliations:** 1 Center NTI “Digital Materials Science: New Materials and Substances”, N.E. Bauman Moscow State Technical University, 2nd Baumanskaya St. 5/1, 105005 Moscow, Russia; 2 Infochemistry Scientific Center, ITMO University, Lomonosova street 9, 197101 St. Petersburg, Russia; 3 Institute of Physiologically Active Compounds, Russian Academy of Sciences, 1 Severniy pr., Chernogolovka, 142432 Moscow, Russia; 4 School of Physics and Engineering, ITMO University, Lomonosov street 9, 197101 St. Peterburg, Russia

**Keywords:** extrusion 3D printing, shear-thinning, hydrogel, polymer composite

## Abstract

In this paper, we report a simple modification of a commercially available printer with fused deposition modeling (FDM) technology for the implementation of extrusion printing of hydrogels. The main difference between an FDM printer and a gel-extrusion printer is their material propulsion system, which has to deal with ether a solid rod or liquid. By application of plastic 3D printing on an FDM printer, specific details, namely, the plunger system and parts of the gel supply system, were produced and combined with a modified printer. Two types of printing of polymer hydrogels were optimized: droplet and filament modes. The rheological ranges suitable for printing for each method were indicated, and the resolution of the samples obtained and the algorithms for creating g-code via Python scripts were given. We have shown the possibility of droplet printing of microspheres with a diameter of 100 microns and a distance between spheres of 200 microns, as well as filament printing of lines with a thickness of 300–2000 microns, which is appropriate accuracy in comparison with commercial printers. This method, in addition to scientific groups, will be especially promising for educational tasks (as a practical work for engineering students or for the introduction of 3D printing into school classes) and industrial groups, as a way to implement 3D extrusion printing of composite polymer hydrogels in a time- and cost-effective way.

## 1. Introduction

Three-dimensional (3D) printing is an additive manufacturing technique where material, or so-called “inks”, are deposited on a substrate via computer numerical control machining [1]. There are plenty of different 3D printing techniques based on stereolithography, laser sintering, inkjet printing and fused deposition modeling (FDM) [2]. In this paper, we focused on 3D extrusion printing, which is a subtype of FDM technology and consists of the extrusion of inks (gel, viscous liquid) in a layer-by-layer way, where fixation of the shape of the final material occurs spontaneously or under external stimuli after contact with the substrate. The advantages of extrusion printing are (i) obtaining materials with complex geometries, [3] which is in high demand, especially for implant fabrication; [4] (ii) carrying out a precision distribution of components in the material both in the form of a pattern [5] or with a gradient distribution; [6] (iii) achieving the alignment of nanoparticles during the printing process under shear stress [7,8] or external field; [9] and (iv) encapsulating cells to mimic physiological environments [10].

Since one of the important applications of 3D extrusion printing is bioprinting, i.e., printing with inks containing living materials (cells, bacteria, cellular spheroids), [11] many modern 3D extrusion printers (CellInk, EnvisionTEC’s, etc.) (https://bioprinting.ru/en/press-center/publications/the-top-15-bioprinters/ (accessed on 1 November 2022)) include elements for sterilization and precision temperature control, which significantly increases their price, but it does not have a critical value for conventional extrusion printing (not bio). Despite the undoubted engineering excellence and scientific importance, such printers remain inaccessible to many researchers (because of the price or the policy of the manufacturer; for example, Organovo’s NovoGen MMX are not made for sale) and even more so cannot be used in educational programs such as in schools, for example.

Considering that FDM printers are among the most common and affordable, [12] while the printing technology is similar to extrusion, it is promising to develop a simple, cost-effective and available modification of the FDM printer for its compatibility with ink for 3D extrusion printing.

For extrusion printing, thixotropic gels or viscous liquids are used as inks at room temperature or with heating up to 60 °C, [13] which is not compatible with commercially available FDM printers that are designed to work with relatively thick solid rods of plastic that than melts at temperatures of 80–280 °C. Thus, the modification consists of forming a new printhead, choosing and engineering a method of material extrusion (pneumatic, screw-driven or piston) [14], and a program supplementary for the new desired behavior. As an additional advantage, we preserve the initial printer’s ability for extrusion printing on plastic, which simplifies its reverse transformation. Therefore, the same printer could work with two different types of materials: both with hard plastic at temperatures >80 °C and with gel/viscous liquids at r.t.

The choice of ink composition for 3D printing is based on the desired properties and applications of the manufactured part [15]. Colloidal thixotropic gels can be used as ink, which is liquified when squeezed out and behaves like a liquid, and when the shear stress is removed (contact with the substrate), they retain their shape, demonstrating the behavior of a solid [15]. In addition, viscous solutions or colloidal systems can be used as ink, where the fixation of the shape can be achieved after printing by applying an external stimulus—temperature, [16] UV irradiation, [17], or immersion in a solution of a crosslinking agent [18]. Printing can be done in drop-by-drop mode to obtain microlenses [19] or microcapsules for drug delivery [20], as well as in layer-by-layer continuous printing mode to create 3D materials, for example, for implants [4]. For each type of printing and ink, it is necessary to select a range of rheological parameters that allows for the achieving of the accuracy of reproducing the desired pattern on the selected type of printer.

In this work we reported a development of a kit of tools for simple, fast and cost-effective modification of a commercial 3D FDM printer for printing hydrogels and viscous solutions for the first time. Also, the proposed scheme is convenient because the main parts are created on the original printer, and consumables (screws, connecting tubes, syringes, needles, etc.) are generally available to individuals. Among different designs of gel supplying, piston-driven, pneumatic and screw-driven, we focus on the first one because of its suitability with drop printing mode. Special nozzle-holders were designed and printed to hold different types of needles. We wrote a number of Python scripts for the most typical printable patterns (drop printing and filament printing) and combined them with open printing software to manipulate the printer. All materials are in open access and readers are encouraged to seek additional clarification.

## 2. Materials and Methods

### 2.1. Materials

#### 2.1.1. Chemicals

Poly(diallyldimethylammonium chloride) (PIL-Cl, high molecular, 20 wt% in water, Aldrich), silver nitrate (99.9%, JSC Bertuz), sodium alginate (Aldrich), calcium chloride (99%, Reagent Component). Commercial fluorocarbon-based solution: Salton.

#### 2.1.2. Materials

Glass syringe (Imi Norgen); dispensing needles with gauge sizes of 14, 15, 16, 17, 18, 19, 20, 21, 22, 23, and 24 were obtained from the AliExpress chuilian official store, Shenzhen Jinguiyuan Technology Co., Shenzhen, China, and sizes 26 and 32G were acquired from the AliExpress Feng Yi Lian Chao Tool Store, Shenzhen Youyi Lianchao Technology Co., Shenzhen, China. PPTF tubes with an inner/outer diameter of 0.5/3.0 mm, 0.3/0.6 mm, 1.0/2.0 mm were purchased from the ShangSuRenPin AliExpress store, Shenzhen City Fangcan Technology Co., Shenzhen, China; the WanPlast PLA plastic came from Moscow local supplier ‘zona-3D’, as did the cylindrical guide rail, guide rail holder, lead screw, bearings, coupler, screws and nuts, and the stepper motor Nema 17 (see Supplementary File S1, Section SI for pictures and comments).

### 2.2. Ink Preparation

#### 2.2.1. Inks for Droplet Printing

An ionic polymer with a nitrate anion, PIL-NO_3_, was synthesized by anion exchange reactions with a silver salt. 1.19 g (0.0070 mol) AgNO_3_ was added to a solution of 1.13 g PIL-Cl (0.0070 mol) in 35 mL H_2_O with vigorous stirring at room temperature. After 2 h, the precipitated AgCl was removed by centrifugation (10,000 speeds per minute, 20 min, 3 times). The resulting polymer was dried in an oven at a temperature of 40 °C for 48 h. Yield: 1.23 g (87%). Anal. calcd for C_8_H1_6_N_2_O_3_ (188.23): C 51.06, H 8.50, N 14.89; found: C 50.01, H 8.63, N 12.95; Belstein test: negative; IR (ATR-mode, intensity: vs.—very strong, s—strong, m—medium, w—weak) (s): 1646 (w), 1473 (m, νCH), 1341(vs), 1045 (w, νCN), 835 (m, νNO); Inks were obtained by dissolving of dry polymer PIL-NO_3_ in deionized water to obtain 20, 15, 10, 5 wt% solutions.

#### 2.2.2. Inks for Continuous Printing

Printing inks were obtained by dissolving sodium alginate in deionized water to obtain 4, 2, 1, and 0.5 wt% solutions. The shape of the printed structures was fixed with a saturated (99.9%) aqueous solution of calcium chloride.

### 2.3. Ink Characterization

#### 2.3.1. Rheological Properties

Rheological properties of inks for drop printing (PIL based) were performed using an Anton Paar MCR 702 (Austria) rheometer with a 1° cone (diameter 50 mm) and plate geometry, and a gap of 100 μm at room temperature (25 °C). To control the temperature, an integrated Peltier plate was used. To measure the viscosity at different shear rates, the systems were equilibrated for 10 min at 25 °C, followed by a flow ramp experiment, in which the shear rate varied from 0.01 to 100 s^−1^. 

Rheological properties for continuous printing (alginate based) inks were performed using a 2° cone (diameter 25 mm) and plate geometry, and a gap of 108 μm at room temperature (25 °C). Shear-thinning (recovery) properties of the system were studied using shear rate oscillation experiments (shear rate in the range from 0.01 to 100 s^−1^). The oscillation time test was taken for 120 s at a strain of 1 % and an angular frequency of 10 rad/s. 

#### 2.3.2. Contact Angle

Wettability measurements were carried out on an Android-based operating system (Android version 7.0 smartphone) app “Dropangle” developed by Guselnikova et.al. [21] as a user-friendly portable module. For the mobile-phone assisted wettability detection, samples were placed parallel to the optical axis in such a way that the cross-sectional view of the water droplet can be captured by the camera in the mobile phone. The measurement algorithm includes the following steps: (a) imaging by using standard camera from a mobile phone, (b) image scaling and translation using the app “Dropangle”, and (c) anchor setting and contact angle estimation using the app “Dropangle”. The contact angle estimation was calculated geometrically by finding the angle Θ from the coordinates of three anchor points, A, B and C. Contact angles were measured using an environmental drop shape analysis system using about 3 μL solvent drops. The average value of the contact angle was obtained by measuring 9 times in 3 different positions for each sample.

#### 2.3.3. Attenuated Total Reflectance-Fourier Transform Infrared Spectroscopy (ATR-FTIR Spectroscopy)

The ATR-FTIR spectra was performed on a Thermo Nicolet iS10 spectrometer with a Fourier transducer using a germanium crystal in the range of 4000–600 cm^−1^ and 30 scans.

#### 2.3.4. Elemental Analysis

An elemental analysis was performed using a LECO TruSpec MICRO elemental analyzer (CHNS) for dried polymer sample.

### 2.4. Coding

Programs for the operation of 3D printing were performed using open software as Python version 3.8 and older, and Pronterface version from 03.02.15. Scripts was written in PyCharm Community Edition, version 2021.0 and older. Two scripts were developed, namely (i) for droplet printing, two files (Supplementary Materials, drop printing scripts); and (ii) a gel form printing rewriter (Supplementary Materials, continuous printing script)

### 2.5. Printer Modification

For 3D modeling we used Fusion 360 software, version 2021 and older, and Cura slicer v.4.3 (both are free for non-commercial use). Any appropriate analogue is allowed. Two blocks were designed and 3D-printed using the initial FDM printer and PLA plastic, namely, the plunger and header for the printing head. Respective 3D models are provided in the Supplementary Information, ‘3D models’ folder. To connect the plunger with the 3D printer body a stepper motor cable extension was used; for connection of the plunger with the header/printing head, a syringe, PPTF tubes and a needle were used (Supplementary File S1, Section SI).

### 2.6. Drop Printing

For a droplet printing a PIL-NO_3_ aqueous solution with concentrations of 5, 10, 15, and 20 wt% were used. The droplet volume of the polymer solution ranged from 12.5 nL to 188 nL. Drops were deposited to substrates of different hydrophobicity (glass, silicon plate, fluorocarbon coated silicon plate) to form a chess-like pattern with a distance between droplets ranging from 2 mm to 500 μm. A 32 g needle was used in most cases, with a 24g needle suitable only for drops larger then 500 μm.

### 2.7. Continuous Printing

For continuous printing, solutions of sodium alginate were used, with weight concentrations ranging from 0.1 to 4 w%. Having adopted the traditional instructions for plastic printing, we created squares with sides of 2 or 4 cm. The infill density ranged from 0 to 90% of empty space in the square. The thickness of the layer varied from 0.1 to 0.3 mm. Thus, the overall demand of inks per square ranged from 10 to 100 microliters. The needle size had to correspond to plans for printing line thickness, therefore needles of 14, 15, 16, 17, 18, 19, 20, 21, 22, 23, and 24 g-size were tested. Once printed, the structure was soaked in a saturated calcium chloride solution to improve its mechanical properties.

## 3. Results

### 3.1. Modification of Commercial 3D FDM Printer

This section is structured in the format: ‘principal scheme—realization—particular parts for realization’, so consequently the idea of modernization and then practical realization are described. To explain this in more detail and comprehensively, the description of all the details needed for the reequipment follows the general description of the gel printer.

#### 3.1.1. Setup

We have developed a set of parts that allows us to modify any commercially available FDM printer for printing with gel-like or highly viscous materials at room temperature. The purpose of this work was to develop a set of universal parts rather than to create completely new or specific equipment, as well as to preserve the possibility of FDM 3D printing on the original printer. Therefore, all parts were designed to be removable, and by installing the appropriate “kit” (for FDM or for extrusion printing), the desired type of material could be used.

**Figure 1:**
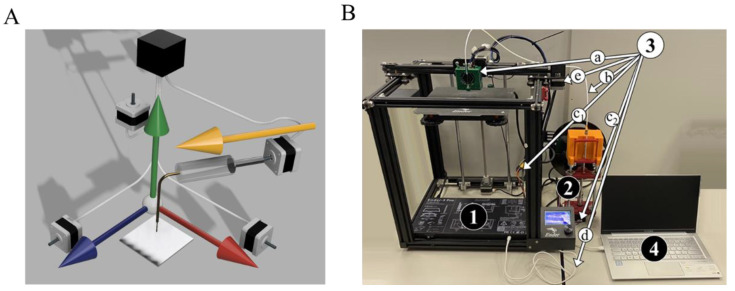
Principal scheme of the modified 3D printer for gel extrusion and its practical realization. (**A**). The scheme represents the general idea of any 3D printer, where the blue, red and green colored arrows represent the relative movement of the substrate-needle, and the fourth yellow arrow is for the filament axis. (**B**). A practical setup for extrusion printing of hydrogels and polymer solutions. The setup for extrusion printing of hydrogels and polymer solutions is as follows: (1) a commercial 3D-printer; (2) a plunger with a stepper motor to push the piston; (3) connection accessories: (a) header as a nozzle holder, (b) pipeline connection syringe to header, (c) re-connection wire to plunger stepper motor, (d) laptop plug, (e) stock stepper motor; and (4) a personal laptop.

All 3D printers are based on 4-axis movement, as shown in Figure 1A. While 3 axis are classical Cartesian coordinates, the fourth axis represents a ‘position’ on the used filament line. The more filament is used, the further the coordinate is, as more steps of a stepper motor are done. The particular type of controllers that are used are not crucial, since all of them are standardized to G-code program language, which is a special tool created for sending precise commands for stepper motors. All axes have a corresponding stepper motor, which via a relevant mechanical joint (not shown) results in strict positioning in space and pumped material. The fourth axis stepper motor is a joint screw-type mechanism that converts rotational movement to propulsion of a piston in a syringe with inks. All stepper motors are connected with white wires to a controller, shown as a black box in Figure 1A. The controller sets have a certain amount of steps in time for every motor, thus crafting a precise movement and dozing. Thus, if we add a coefficient to change the steps of the initial plastic filament propulsion to steps of piston propulsion, we can preserve the structure of classical 3D sliders, and thus any 3D software is potentially usable. To sum up, the slicer writes commands to four stepper motors to make steps, which with our corrections results in positioning and screw rotation, leading to piston propulsion of the plunger supply system.

**Figure 2:**
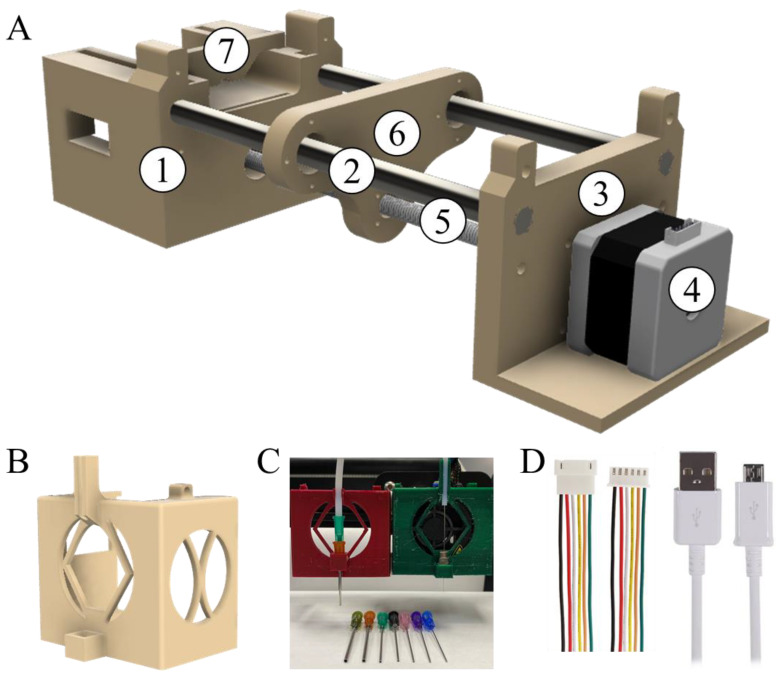
Modification parts, both engineered and commercially available, to reequip a standard 3D printer to deal with liquid and gelled inks. (**A**). Plunger supply system: FDM parts of construction: 1, 3—construction holders, 6, 7—pusher and syringe holder; CNC parts: 2—8 mm guide rails, 4—Nema 17 stepper motor, 5—lead screw rod. Metal locks and bearings are omitted for visualization simplicity. (**B**). Printed holder of needle and tube (nozzle holder), (**C**). Examples of tube connection of different inner and outer diameter, (**D**). Wire connections of printer with stepper and printer with laptop (Figure 1B, c,d). Note: the printer is not to be used in autonomous mode, as this could potentially damage the equipment.

**Figure 3:**
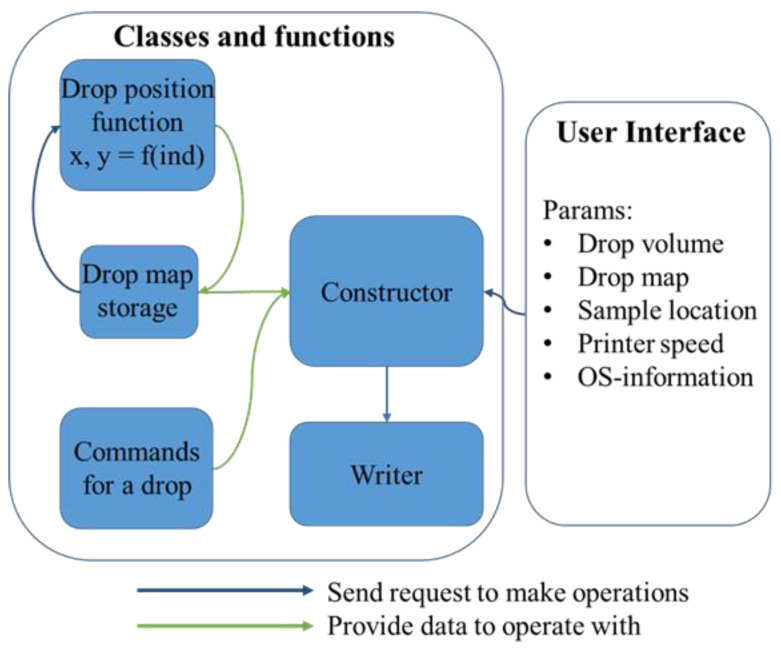
The flowchart of data processing while creating g-code instructions. The blue and green arrows represent calling for and providing information, respectively. In User Interface (UI)-script one enters the parameters of printing. This script is called the operational script “Classes and functions”. The constructor class get the params from UI, and start creating a list of instructions for the 3D printer. It takes the drop coordinates from Drop-map storage, which via position function, returns list of drops’ coordinates. After processing each drop with suitable commands, Constructor sends data to the writer, by calling it. The writer then creates the g-code file.

The realization of the scheme on Figure 1A applied to a commercial 3D printer has for main blocks: (i) a commercial 3D-printer (we used a Creality Ender 5 pro, but the model is not crucial) (Figure 1B, 1); (ii) a plunger with a stepper motor, (Figure 1B, 2); connection accessories including a header, supply pipeline, and wires, (Figure 1B, 3a–d); (iv) and a laptop with open-access program software (PyCharm, Pronterface, Cura, Fusion 360). We design both the header and plunger, while connection parts such as PPTF tubes, syringes, and cables are commercially available. The header (3a) is used as a nozzle carrier and allows for simple nozzle changing. This part could be model-specific for certain 3D printers, but it is easy to redesign it and then print a new one. We note that the stock FDM equipment of the printer is unchanged, and could be used in a classical way by simple removing our printing head and plunger. In particular, the printing head (Figure 1B, 3a) has to be removed, and a wire (Figure 1B, 3c) has to be reconnected from the plunger stepper motor to stock one (Figure 1B, e). Among other engineering issues, the stock tube connection has a huge dead volume of approximately 5 mL, while the average gel consumption was up to 100 µL per print. Finally, the nozzle change for FDM is tricky and demands heating up to 150–200 degrees Celsius, and does not permit any needle to be equipped.

#### 3.1.2. Plunger

**Figure 4:**
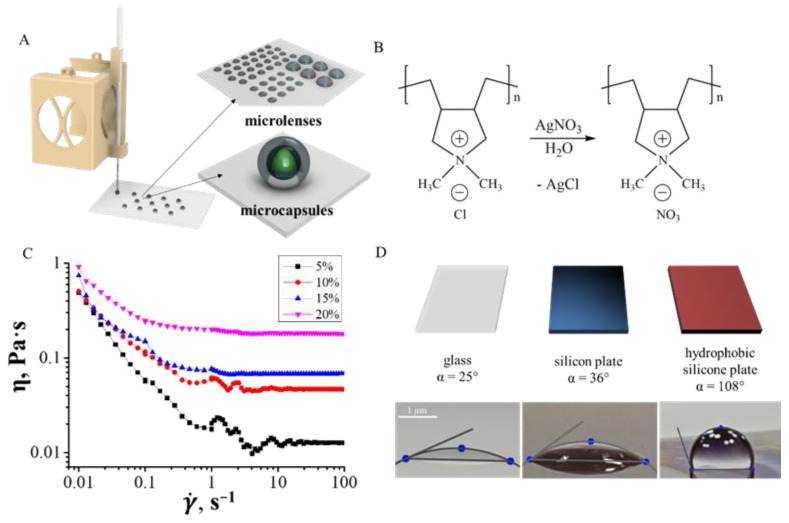
Scheme of droplet printing, ink parameters and substrate options for microlens printing. Ink parameters for droplet printing. (**A**) a schematic representation of the principle of droplet printing and possible applications of printed structures, (**B**) a scheme for the synthesis of an ionic polymer PIL-NO_3_, (**C**) variation in shear viscosity plotted as a function of the shear rate for inks with different concentrations PIL-NO_3_ (5, 10, 15 and 20 wt%); (**D**) top: scheme of used substrates, and bottom: contact wetting angle with glass, silicon and hydrophobized silicon.

**Figure 5:**
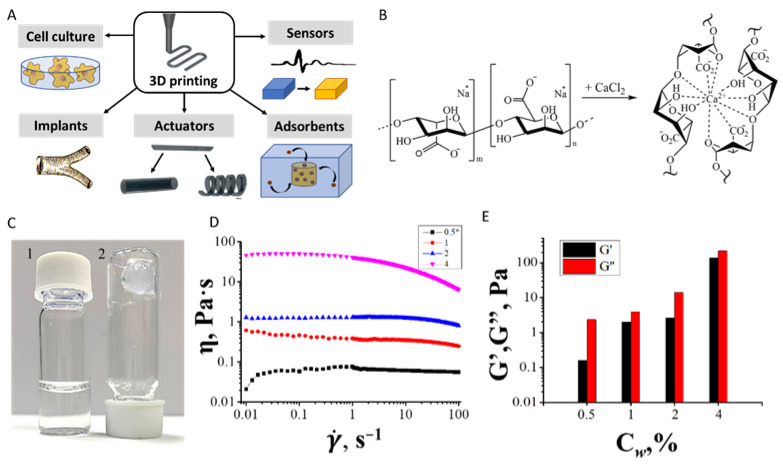
Scheme and application of continuous printing and its ink parameters of continuous printing. (**A**) gel printing sample and examples of the application of printed structures, (**B**) scheme of gel formation during treatment of sodium alginate with a concentrated solution of calcium chloride, (**C**) example of a alginate-based inks before (left) and immediately after (right) treatment with calcium chloride, (**D**) variation of ink viscosity from 0.5, 1, 2 and 4 wt% alginate depending on the shear rate, (**E**) storage (G′) and loss (G′′) moduli of sample containing alginate 0.5, 1, 2 and 4 wt%.

**Figure 6:**
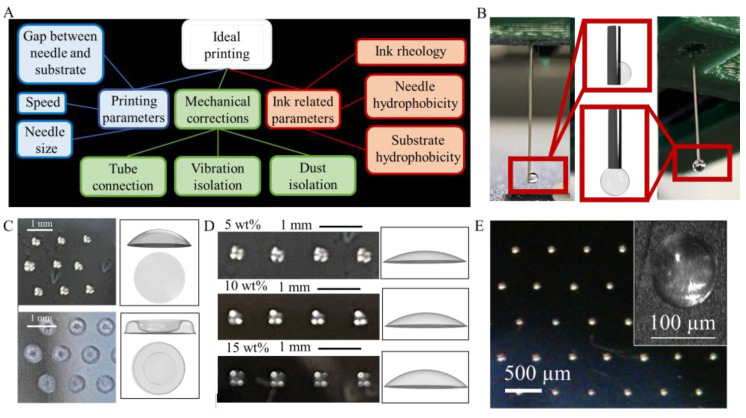
Tunable printing parameters and their influence on dropwise printing quality and the final result. (**A**). Parameters for droplet printing to be monitored. (**B**). drop of hydrophilic ink formation on hydrophilic needle (left) and on hydrophobic needle (right). (**C**). Spherical dome formed on hydrophobized silicon, toroid structure of coffee-ring on pure silicon. (**D**). Spherical dome of different curvature due to different polymer concentration in inks. (**E**). Example of well-tuned microlens printing on a hydrophobic surface with a hydrophobic needle.

**Figure 7:**
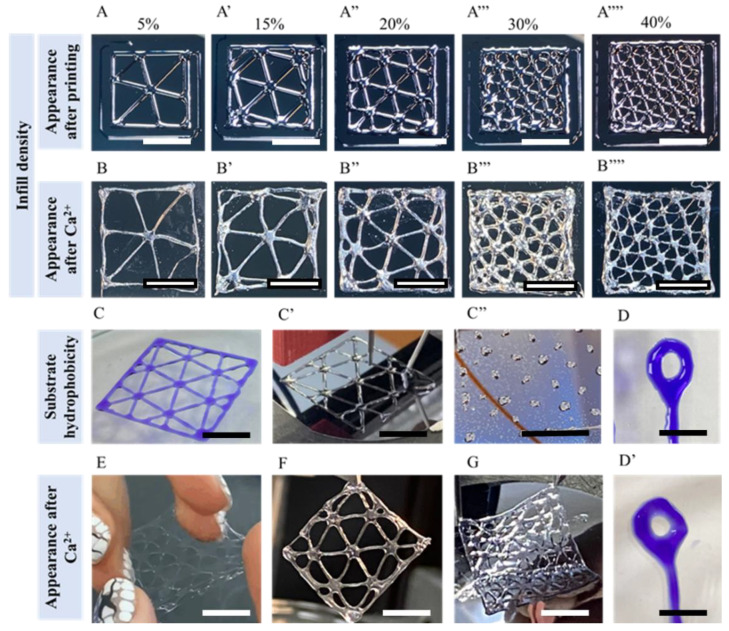
Tunable printing parameter, its consequences and possibilities of continuous printing. (**A**) 2 × 2 cm printed square with infill parameter ranging from 5 to 40% on a pure silicon plate, using a g24 sized needle, 4 wt% alginate based inks and the skirt line around a square to stabilize the ink flow. Scale bar is 1 cm. (**B**) the same objects from (**A**), but treated with calcium chloride saturated solution. Scale bar is 1 cm. (**C**) 4 × 4 cm square printed with 4 wt% alginate inks using a g20 sized needle on a different types of surface, from left to right: glass as hydrophilic surface, silicon plate as slightly hydrophobic surface, a fluorocarbon coated silicon plate as a highly hydrophobic surface. Scale bar is 2 cm. (**D**) printed complex shape of closed loop 4 mm in diameter, inks with a blue dye, (**D’**) the same after hardening in calcium salt solution and capable of carrying a load. Scale bar is 0.5 cm for (**D**) and (**D’**). (**E**–**G**)squares of different infill, hardened and capable to carry a load. The scale bar is 2 cm.

One of the key components for the transition from FDM to gel-extrusion printing is a piston pump. While there are commercially available ones, their synchronization with the FDM printer is limited, since mechanical connections could not be replaced (for instance, the equipment case tends to be a monolith, so it is hard to make any mechanical changes) and program software is not open access (the code is owned by the supplier). Another advantage of our approach is the cost-effectiveness of the formed plunger. Thus, we engineered a plunger with commercially available accessories and 3D-printed parts (Figure 2A). Hence, the printed body (Figure 2A, 1) is joined with a stiff metal guide rail (Figure 2A, 2) with a printer platform (Figure 2A, 3) that holds a stepper motor (Figure 2A, 4). The stepper motor drives a lead screw gear (Figure 2A, 5) that pushes a plunger platform (Figure 2A, 6). Finally, a syringe is fixed on a body (Figure 2A, 1) with printed holders (Figure 2A, 7). The particular sizes of the rods are not crucial; we used an 8 mm rod as the most common one. However, we would not recommend using rods thinner than 6 mm (or one has to redesign the plunger for higher rigidity). Further information is provided in Supplementary File S1, Section SI.

On Figure 2A, standard bearings and rail holders are omitted, as these parts could differ in different shops and countries, while it is easy to redesign a 3D printed part with most available accessories.

The minimal printed volume of this plunger system depends on stepper motor characteristics, such as the lead screw step, the amount of threads and the piston diameter of the syringe. In particular, we were focused on the following parameters: the minimal rotational degree of the stepper motor (we used standard 200-steps motor, i.e., 1.8 degrees), the thread lead, and the syringe piston surface. Consequently, the minimal step of the stepper motor and the lead combined results in minimal piston movement Δh, which, being timed by piston surface S, results in minimal volume of ΔV = Δh · S ≈ 125 nl for high viscous liquids (more details are provided in the description of code parameter drop volume). However, the precision of the material deposition of the setup is determined not only by plunger characteristics, but also by the quality of the connections between plunger and nozzle. Air bubbles, leaks, and high elasticity of the tube connection result in a slow pressure wave, leading to printing imperfection, and thereby have to be avoided. To achieve that, one has to follow the following advice: (i) degas a printing solutions, (ii) when connecting tubes and needles be sure to prefill cavities with the printing solution via syringe even with a thinner needle, (iii) use tubes with thick walls, a 1.25 mm of PPTF capillary is sufficient, (iv) choose a needle with an outer diameter bigger than inner diameter of tube at least by 0.2 mm and a depth of connection of about 1 cm, (v) avoid sharp corners in the pipeline, and (vi) make the pipeline as short as possible while remaining sufficient for one’s tasks. 

#### 3.1.3. Connections

We choose to separate the printhead and plunger pump in order to avoid the excessive mass of moving parts. That decision let avoid several issues, namely (i) the weighting of the printhead requires strengthening the structure of the original 3D printer, and (ii) the high vibrations upon acceleration of the heavy printing head, which reduces the accuracy of the deposition of the material. This way, the printer precision in absolute positioning is of 100 microns error bar, while the error of distance between droplets is within 10 microns. The minimal distance between drops also depends on the needles used, and in our case the best option was 250 microns. The features for drop volume are described below.

The disadvantages of the separation of the printhead and plunger pump include the requirement to use longer connecting tubes, which leads to pressure waves. Stiff tubes with thick walls prevent balloon-like inflation, thus reducing wave amplitude. Another important option is the prevention of leakage. That is possible by tight connections from tube to needle, and a needle has to be 0.2 mm thicker than inner diameter of the tube. A 23 g needle works well for a capillary with 0.5 mm of inner diameter. In case one needs to use a thinner needle, it is a good idea to glue a 23 g needle to a32 g one. While using epoxy, it is important to fit needles well and avoid getting air in the resin. Thus, to achieve high precision and reproducibility for the printing of gels it is important to use stiff non-elastic tubes and to avoid air bubbles within the linkage. That is why PPTF tubes with a wall thickness >1 mm and degassed solutions are highly recommended. Also, any pipeline kinks have to be avoided.

To connect all the bodies into one functional piece of equipment we need three elements: (i) a nozzle holder, (ii) a nozzle with hollow PPTF tubes/capillaries, and (iii) two wire plugs. The nozzle holder is modeled to be removable, and is located on the print-head (Figure 1B, 3a and Figure 2B,C)). Any needle or glass capillary could serve as a nozzle, which is then connected to syringe via a stiff PPTF tube in a replaceable way (Figure 2C). A wire connector extension (Figure 2D, left) was used to achieve the easy reconnection of the stepper motor that is used for plastic to the gel-one. To connect laptop and the printer we use a stock mini-USB wire, as shown in Figure 2D, right.

It is important to highlight that although we minimize pressure waves by stiff connections and the degassing of solutions, the complete elimination of them is physically impossible. That is when we consider two special code tricks that either for droplets or for continuous printing allow us to avoid the lag of gel propulsion. They are waiting time after every drop and draft line called ‘skirt line’ printed before main print, and they are described in detail in the corresponding sections.

#### 3.1.4. Coding

The general idea of Python coding here is to create a text file with set of G-code instructions. That could be done either by rewriting the file that was created by special 3D programs, which is useful for continuous printing, or “from scratch”, as in the case of droplet printing. We note that the first method involves the simple linear changes of values in existing files, which is of scientific or programming interest. The second way, by write commands from scratch, is more complicated, but the main idea does not change: we take positioning and pumping parameters and algorithmically create G-code instructions for the 3D printer.

The code for droplets printing was written in Python 3.8 and consists of two files: (i) file of user interface (UI) and ‘Classes and functions’, which is a calculation file (Figure 3; Supplementary File S1, Section SII).

The UI is a place where parameters (or params) of the printing should be entered. These are (1) drop volume, (2) parameters of drop location (or “drop map” parameters), (3) sample location on a printing table, and (4) printer speed and operating system information.

(1) Drop volume, a parameter applied for every drop is not a straight value, and is measured in arbitrary units, one of which is equal to 0.1 π of the lead screw rod (Figure 2A, 5) rotation. The screw rod rotation transforms to linear motion of the syringe piston, and the lead (i.e., the coefficient of transformation from one full rotation to linear motion) depends on the number of thread systems and thread pitch (thickness of thread). In our case, the lead is equal to 2 mm, thus 10 au results in 1 mm of piston propulsion (in forehead expression set as ‘l’). The final volume, V, should be calculated based on piston radius, r, according to the Equation (1):

V = π · r^2^ · *drop_volume* ·10; [V] = mcl, [r] = cm. (1)

The 500 mcl syringe we used has a piston diameter of 4 mm, or r = 0.2 cm. The motor has 200 steps per 2Pi, thus 0.01 Pi or 0.1 au is the minimal whole step. Thus, the volume is 3.14 × 0.22 × 0.1 × 10 = 0.1256 mcl or 125.6 nl. However, there are partial steps, so-called “microsteps”. The amount of microsteps is strictly regulated by internal printer firmware and is generally equal to 16, i.e., every single step could be splitted into 16 more equal microsteps. In such a case, the minimal volume is equal to 0.00625 au or 7.85 nL. There is a drawback to this method: the torque of motor decreases drastically upon microstepping. The smaller the microstep is, the bigger the loss of propulsion force is. For 1/16 of step the propulsion force is about 1/10 of full step force. That is why when one uses a very viscous liquid and a drop_volume of less than 0.1 au, it is obligatory to check if the motor has enough torque to deal this task by just watching if the drop formation is straight and reproducible.

Overall, we set the rotation that defines the linear propulsion of piston, in this way creating the drop volume.

(2) The ‘Drop map’ is a block of parameters which correlates with internal function ‘location_function’ in the calculation file of the code. In our case, to describe a drop location map, one should enter the amount of columns and amount of rows of a chess-like map, and set the horizontal shift and vertical shift correspondingly. Overall, these four parameters are combined in Figure 3 as ‘drop map’. If a more complex pattern is needed, the location_function has to be changed in both of its parameters.
(3) *Starting_point_coordinates_x_y* and *z_coordinate_when_touching_pattern* are straight values that state the initial position of a sample on a table. Coordinates of sample location on the printing table are entered in pair (x, y), and separated z as a height.
(4) *Printer speed* is a straight value that shows is the maximal achievable amount of mm per second for the printhead. The real speed of the printhead is affected by two more sets of parameters, particularly acceleration and jerk. Acceleration states how fast the changing of the printhead occurs, and the jerk is maximum instantaneous velocity change. For that, we set limits on the jerks and accelerations in the Constructor block, and these parameters are not implied to be changed. We set them to achieve maximal smoothness when the jerk for movement is 0, so no sharp movement is allowed, and the acceleration is of a relatively small value of 500 mm/s^2^ in order to minimize vibrations (ten times less than for average printing). The operating system information consists of two variables: the filename and address (folder in C:/). If file with such a name exists, it would be overwritten.

The second hidden from user block, which is ‘Classes and functions’, operates on the parameters from UI and creates a file in ‘.gcode’ format (this is the standard format for CNC machining and 3D printers in particular)., This block contains the Coordinates class, the GCodeInstructions class and ‘name_of_function’. The first class, Coordinates, takes user input from the UI file and returns a list of coordinates. This class possesses several functions to produce coordinates for droplets. In the case of new specific patterns to print one has to create a new function in this class. Once calculations are done, the list of coordinates is transferred to GCodeInstructions class. There the coordinates and user instructions are merged in a demanded way, and the result is a list of g-code instructions for the printer. That class is responsible for changing the initial positions or printing conditions. To achieve a new pattern while printing, that class has to be updated. The current behavior is straightforward and quite simple. 

In particular, the printing process is split into three different processes: moving toward a point, pumping a droplet, and a touch-&-back move. While an attempt to join these steps into one leads to faster print, it also results in a changed position or size of the printed drops. The instructions for drop processing are summarized in ‘Command for a drop’ block. There we wrote set of commands to achieve the highest precision of the print of ±10 microns.

Since it is physically impossible to eliminate the lag of pumping completely, there we introduce a fixed waiting of time half a second for the fixed waiting time. This parameter is written in instructions of drop processing and does not need to be changed. However, if one is sure it is necessary, it is possible just by changing it inside “commands for a drop”. Once the G-code instructions file is created, one should transfer it to printer via Pronterface. That software is comfortable to monitor the printing process.

### 3.2. Inks Rheology

#### 3.2.1. Inks for Droplet Printing

In droplet printing, a polymer solution is supplied intermittently to form drops, which are deposited on the surface, and, when the solvent evaporates, form a certain geometry: spheres, hemispheres or coffee rings. Depending on the geometry and nature of the ink, it can be used as microlenses [19], or microcapsules [22] (Figure 4A). In drop-by-drop printing, it is important to control the viscosity of the ink and the wetting angle with the substrate on which the droplets are deposited. The smallest droplet size determines the resolution of the method (usually on the order of 10–100 µm). Here, we describe droplet printing using an example of an ink based on an aqueous solution of an ionic polymer (Figure 4B) for application as microlenses (hemisphere shape). Thus, the rheological properties were adjusted only by variation of polymer concentration and no surfactants were added, because they can affect the optical and physical properties of the printed object.

Figure 4C shows the change in shear viscosity of an ink based on an aqueous solution of polydimethyldiallylammonium nitrate (PIL-NO_3_) with different mass concentrations of 5, 10, 15, 20 wt% depending on the shear rate. All inks demonstrated shear-thinning behaviour, i.e., a decrease in viscosity with increasing shear rate [5]. The viscosity of the ink for concentrations 5, 10, 15, 20 wt% at 0.01 s-1 shear rate was 0.49, 0.50, 0.74, 0.92 Pa·s, respectively, and with an increase in shear rate up to 30 s^−1^, while the viscosity for these concentrations was 0.01, 0.05, 0.07, 0.18 Pa·s, respectively.

The wettability of the substrate by inks affects the resulting shape of the printed object. Figure 4D shows photographs of the contact angle for hydrophilic inks on the substrates with different contact angles: on glass, 25°, on silicon substrate, 36°, on hydrophobized silicon substrate, 108°. Using the example of printing microlenses from PIL-NO_3_, we have shown that by varying the hydrophobicity of the substrate, we can obtain different contact angles and, in the future, different shapes of printed drops. In this example, we wanted to achieve the formation of a hemisphere, so we chose a hydrophobized silicon substrate, since this substrate showed the highest contact angle of 108°.

#### 3.2.2. Inks for Continuous Printing

Filamentary printing is a printing method in which a continuous flow of filament forms 3D structures that are used in the formation of various types of functional materials, actuators, sensors, healing dressings, absorbents, and cell culture matrices (Figure 5A) [23,24,25].

In our work, we took sodium alginate as an example for continuous printing visualization, which, when treated with concentrated calcium chloride, forms gels, which makes it easy to form 3D objects. Figure 5B,C show the scheme and process of gelation based on sodium alginate. By dissolving sodium alginate in water, an aqueous solution is obtained in which the forming alginate units (mannuronic and guluronic units) present their carboxyl groups in anionic form as carboxylate negative ions. The gelation of alginate was accompanied by the formation of a complex of alginate with calcium. The ionotropic gelation of sodium alginate with calcium cations is conditionally described by the “egg-box” model, where calcium cations interact with guluronic acid monomers in cavities formed by pairing of G-sequences of alginate molecular chains (Figure 5B) [26].

Figure 5D shows the change in shear viscosity of an aqueous sodium alginate ink with different mass concentrations of 0.5, 1, 2 and 4 wt%, depending on the shear rate. The alginate-based gel did exhibit thixotropic properties; however, a significant drop in viscosity was observed above a shear rate of 100 s^−1^. The viscosity of the ink for concentrations 0.5, 1, 2 and 4 wt% at 0.01 s^−1^ shear rate was 0.055, 0.62, 1.29 and 46.4 Pa·s, respectively, and with an increase in shear rate up to 100 s^−1^, the viscosity for these concentrations was 0.056, 0.25, 0.81, 6.36 Pa·s, respectively. The study of the storage moduli (G’) and losses (G’’) of the sample containing alginate 0.5, 1, 2 and 4 wt% demonstrate that alginate-based inks are viscous liquids, but not gels (Figure 5E).

### 3.3. Extrusion Printing of Hydrogels

#### 3.3.1. Droplet Printing

The optimized conditions of droplet printing process make it possible to print droplets with a diameter down to 100 μm and a distance between droplets down to 250 μm. The printing quality of a sample is a result of set printing parameters, printer installation and inks design (Figure 6A).

Ink-related parameters include ink rheology and ink interaction with substrate and needle. We tested viscosity for PIL-NO_3_ aqueous solutions with concentrations of 5–15 wt%, and sodium alginate aqueous solutions with concentrations of 1–4 wt% (Figure 6C). All polymer-based inks were possible to pump through the set up. However, inks with the highest viscosity at shear rate 30 s^−1^, namely 4 wt% alginate-base ink (viscosity 12.5 Pa·s) and 20 wt% PIL-NO_3_ ink (viscosity 0.18 Pa·s) resulted in irremovable bubbles inside the solution, leading to clogging. Upon further increasing viscosity, one finds two viscosity thresholds (one when the minimal step becomes 125 nL, another when the printer halts), but since bubbles already have disruptive consequences, we did not investigate more viscous values. 

The interaction of ink with a needle influences the positioning accuracy, or even prevents the possibility of drop deposition. Both the needle and the working solution was highly hydrophilic, and a drop spread upward to cover the needle (Figure 6B, left). To prevent that, we covered the needle with a commercial fluorocarbon hydrophobization solution to make it hydrophobic, and the resulting shape is a sphere (Figure 6B, right). Thus, the opposite hydrophilicity of a needle and a working solution is highly recommended for a reproducible drop formation.

The hydrophobicity of the substrate influences both the form of a deposited drop and the final shape of the dried ink droplet. For ink with 15 wt% PIL-NO3 solution, the contact angles were tested on glass, pure silicon, and hydrophobized silicon, giving angles of 25°, 36° and 108°, respectively (Figure 6C). Upon drying, the ion polymer solution on the hydrophobized silicon formed a spherical dome (Figure 6C, top), while on pure silicon a coffee-ring effect [27] governed by Marangoni flow lead to the formation of a toroid structure (Figure 6C, bottom).

By variation of polymer concentration in the ink, it was possible to change the height of the obtained hemisphere within one type of substrate (Figure 6D). Therefore, by using a highly hydrophobic substrate and high percentage of dry material in ink, micro lenses both small in diameter and with high curvature were obtained (Figure 6). 

Printing parameters for high-quality printing include a gap between the needle end and the substrate surface, the needle size and the speed of printing. The gap between the needle and the surface affects print reproducibility and drop size. If the gap between the needle and the substrate is too small (the specific value depends on the size of the drop and the size of the needle), the drop is forced to flatten upon touching the surface, which leads to a larger diameter and a lower height of the resulting hemisphere. If the gap is too large, the droplet deposition may not occur or will occur partially; therefore, the microlenses will be of different sizes. In our setup, the optimal gap was 0.05 mm for a drop volume of ~15 nL, 0.1 mm for ~75 nl and 0.2 for 150+ nL (needle size was G32). 

The speed and acceleration parameters determine the self-vibrations of the printer. Overall, with the increase in printing speed, the positioning of the material becomes less accurate and the printer vibration becomes larger, which leads to inhomogeneous printing and inaccurate reproduction of the designed pattern. In our setup, the upper limit for printing speed was 500 mm/min, and the acceleration was of the stock printer values (typically it is about 5 or 10 mm/s^2^).

The needle size affects the size of the resulting droplet. In general, a smaller needle makes it possible to reproducibly print smaller microlenses. However, if the needle is too small for programmed behavior, the drop could fall before the planned point. In particular, we used G32 size of the dosing needle (0.1 mm of inner diameter and 0.23 mm of outer diameter). Our recommendation is to avoid the usage of a needle for drops that more than twice as bigger as needle outer diameter.

Figure 6B represents the best achieved result for printing microlenses with inks based on PIL-NO_3_ of 15 wt% concentration, on silicon plate and 32 g needle are covered with fluorocarbon, drop_size parameter equals to 0.015 a.u. (corresponds to 18.8 nL).

#### 3.3.2. Continuous Printing

3D printing involves the creation of a pre-designed 3D object formed by filaments by deposition of them in a layer-by-layer way. The design of printed patterns can be created in any 3D modeling software, for example by using tinkercad.com. Detailed instructions for entering the pattern and an explanation of the code are contained in Supplementary File S1, Sections SIII–SIV. In short, the script takes all values in already created files and recalculates them as y = kx + b with parameters set by the researcher. The script makes a copy of the instruction file with the newly calculated values.

A standard way to present printing quality is the creation of patterns with different infill density. As an example, we printed squares of 2 × 2 and 4 × 4 cm size with variation of infill density from 5 to 40 % (Figure 7A–A’’’’) using a 24g sized needle and 4 wt% alginate solution.

Patterns made of viscous liquids or gel are not strong, and could be blurred as a result of time, vibrations or mechanical shifts. To preserve the shape of the printed pattern it was covered with saturated calcium chloride solution, which acts as a crosslinker and preserves the transition of a pattern to droplets Figure 7B–B’’’’, E–G.

In addition to the case of droplet printing, we have three groups of parameters that affect the quality (Figure 6A), but in this case needle hydrophobicity is not crucial since a constant flow of the polymer solution is used, and the needle size is recommended to be close to the planning printing line width. Thus, the reproduction quality of the designed pattern (print accuracy and reproducibility) is influenced by the rheology of inks, the printing speed and the hydrophilicity of the substrate. We tested sodium alginate solutions from 1 wt% to 4 wt%, and the latter is about the highest possible concentrations, and that corresponded to viscosity 46.4 Pa·s at shear rate 0.01 s^−1^, viscosity 12.5 Pa·s at shear rate 30 s^−1^ and viscosity 6.36 Pa·s at shear rate 100 s^−1^. All concentrations were easily pumped through, but at 4 wt% it takes a significant effort to eliminate all air bubbles from the ink solution, and air bubbles cause clogging. This is possible to overcome by using a so called ‘skirt line’ close to the printing pattern (see Figure 7A–A’’’), which is practically a line drawn with inks before the main pattern. During this additional line, the pressure inside pipeline stabilizes, allowing the ink flow to be constant and consistent. Inks with a concentration of 4 wt% provide us with the most sustainable print and few parasite flows, thus this concentration was used for most printings. 

Printing with hydrophilic inks on surfaces with high hydrophilicity, such as glass, leads to the intense spread of printed lines, with forehead flow of the liquid to form wide lines and agglomerates (Figure 7C). The best option for printing hydrophilic inks was on a slightly hydrophobic surface, as pure silicon, which lead to formation of homogenous smooth lines (Figure 7B’). Printing on surfaces with high hydrophobicity (hydrophobized silicon) leads to the splitting of printed lines into individual drops that are randomly sized (Figure 7B’’), which is not suitable for continuous printing due to disruption of pattern and not suitable for droplet printing due to the random size of the obtained droplets. 

It is possible to print any desirable shape or form, as we show in Figure 7 D,D’, where we first printed a hanging loop, and then hardened it in calcium chloride solution. 

Printing multilayered structures with semiliquid inks is a tricky and complex task, which we plan to cover in forthcoming work. However, here we have presented a short, comprehensive overview. Thus, the main challenge to be overcome for multilayer structures is to prevent the collapsing or spreading out of the newly printed semiliquid layers. The key role there is to provide a strong underneath for the newly printed layer, in other words, to solidify already deposited semiliquids to strong gels before new layer deposition.

Thus, we have tried three common ways: chemical treatment, UV irradiation and rapid temperature decrease. The method chosen for solidification depends on the experiment design and is the decision of the researcher.

For chemical treatment procedures we used paired sodium aliginate – calcium chloride solutions. Layers of printed polymers were treated with calcium chloride aerosol, obtained from an ultrasound source. The use of aerosoled solidification solution is preferable, since layers could not be washed out, and since it is possible to get the surface of the layer to only be partially solidified. As a result, the layer is already strong enough to carry the load of the new deposition, and at the same time is still reactive to form molecular connections with newly deposited layers. As for UV irradiation, it is important to design inks capable both of partial solidification and extralayer bond formation, otherwise the stracture would delaminate. The drawback of both chemical treatment and UV irradiation is that printing has to be paused and then renewed in order to properly carry them out. We have also printed a 7 layer structure with 5% infill density (Figure S1, Supplementary File S1) and examined its mechanical properties (Figure S2, Supplementary File S1). To improve the printing quality we carried out printing at low temperatures by using a polished solid metal cube frozen to −20 °C which was cooled by storage in a refrigerator. When printing on its surface (optionally, any substrate with a commercial thermal paste and located on an ice pack is appropriate), the gel quickly decreased in temperature and formed a strong printed line.

Since this is less precise compared to when microlens patterns are used, much faster printing is possible with a speed up to 3000 mm/min. The optimal printing speed for 4 wt% alginate inks was 1500 mm/min with standard Cura coefficients (printer speed consists of more than 20 parameters, and Cura software is able to set them automatically in correspondence with the main speed).

## 4. Conclusions

We have developed a simple way to re-equip a standard commercial 3D FDM printer to deposit liquids or thixotropic gels at room temperature by only using 3D modeled and printed parts and some commercially available and cheap parts for computer numerical control machining. The modified printer additionally has a header for the printhead, a plunger pump, wire and tube connections, and three Python scripts. 

We proved the ability of using inks with a viscosity up to 0.74 Pa·s at a shear rate of 30 s-1 for droplet printing; the typical print speed is 1–2 microlens per second. The drop positioning accuracy is ±5 microns, the minimal drop distance is 200 microns, a spherical dome and toroid coffee-ring structure is possible, and the typical size of a microlens is 100–500 microns. This accuracy was achieved by tuning the needle type and positioning and adjusting the substrate hydrophilicity and ink rheology. 

The possibility to use inks with viscosity of up to 12.5 Pa·s at a shear rate 30 s^−1^ for continuous printing is shown. Overall, the basic rules for high-quality printing are the same as for the droplet printing of microlenses. Thus, the high hydrophobicity of the substrate has to be avoided, and the best option is a slightly hydrophobic surface; the system is sustainable for air bubbles inside the ink in case an additional stabilizing line print is used before the main print. Much faster printing is possible with a speed of up to 3000 mm/min. The gap between needle and surface, the needle’s outer size and material flow together determine the thickness and the height of the printed lines. The typical line width is 0.3–2 mm, and the error bar positioning is half of the line width. 

Thus, the proposed scheme is a simple, inexpensive and convenient solution for modifying the FDM extrusion printer for printing gels. The advantage is that the main parts are created on the original printer, and consumables (screws, connecting tubes, syringes, needles, etc.) are available for purchase by individuals. Moreover, the achieved resolution of printing is comparable with commercial printers. The proposed modification will be of interest to both research groups due to the fairly good accuracy of pattern formation, and educational groups due to the simplicity and cost-efficiency of the proposed printer design.

## Data Availability

If readers would like to change 3D files, they can use Fusion 360 public link or redraw them. https://a360.co/3DZhxBj (accessed on 2 December 2022) for plunger, https://a360.co/3UNDUkg (accessed on 2 December 2022) for header.

## Supplementary Materials

The following supporting information can be downloaded at: https://www.mdpi.com/article/10.3390/polym14245539/s1.
