# Supplementary material for: Modification of Commercial 3D Fused Deposition Modeling Printer for Extrusion Printing of Hydrogels
Source: Polymers (Basel). 2022 Dec 17;14(24):5539. doi: 10.3390/polym14245539 (PMC9784586; doi:10.3390/polym14245539)
Supplement: Supplementary file 1 [file polymers-14-05539-s001.zip › decription.docx]

**V. List of files enclosed**

| **File** | **Comment** |
| --- | --- |
| Appendix 1. Drop printing python scripts | Python scripts for creation of droplet, zip archive |
| Appendix 2. Continuous printing python script. | Python scripts to rewrite gcode instructions for continuous printing modes, zip archive |
| Appendix 3. Git-hub link | The whole project in internet source to download.  LINK |
| Appendix 4. Cura project | Saved parameters for linear printing in Cura slicer. This file is not necessary, but recommended in case you are new in 3D printing. |
| Appendix 5. Squre.stl | 3D model of square with thickness of 2 mm. It is used to be imported in Cura for further slicing. |
| Appendix 6. Squre.gcode | Previous file sliced. Implied to be fed to python script. Also suitable for classical 3D printing. |
| Appendix 7. Squre_first_slice.gcode | The g-code instructions after python script is done. Suitable for particular printer with particular sample in particular location. |
| Appendix 8. Stl files for plunger and printer | All files that has to be printed on stock FDM 3D printer in order to re-equip it with them. Zip archive. |
| Appendix 9. Video | Examples of continuous printing with different infill on different types of surfaces, particularly pure and hydrophobized silicon.  Example of drop printing, with experimental and stable mode (specific complex and dome shape of drop correspondingly) |
