# Supplementary material for: Modification of Commercial 3D Fused Deposition Modeling Printer for Extrusion Printing of Hydrogels
Source: Polymers (Basel). 2022 Dec 17;14(24):5539. doi: 10.3390/polym14245539 (PMC9784586; doi:10.3390/polym14245539)
Supplement: Supplementary file 1 [file polymers-14-05539-s001.zip › polymers-2058878-supplementary3.pdf]

Supplementary information

Modification of commercial 3D fused deposition modeling printer for extrusion printing of hydrogels

Koltsov S.I.[1,2]* Statsenko T.G.[1,3], and Morozova S.M.[1,4]*

Kit parts (all parts demanded for the equipment)

Name	Amount, additional comment	Photo
Stock 3D printer	1.<br><br>Any FDM is suitable, stiff frame is preferable. We use Creality Ender 5 Pro.	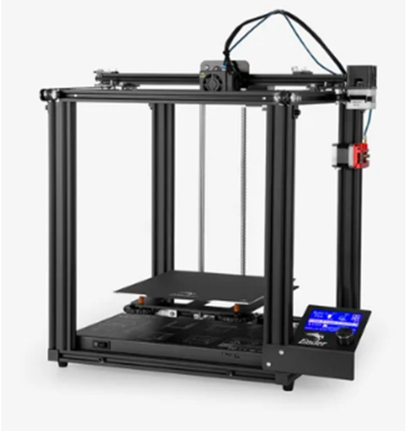
Nema17 4 wire Stepper Motor Cable with 4 Pin Dupont 6Pin HX2.54mm JST Connector	1	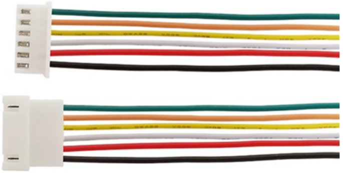
Laptop	1,<br>Any with Windows 7 or higher or Linux.	
Nozzle holder	1+,<br>Designed and printed additionally by demand.	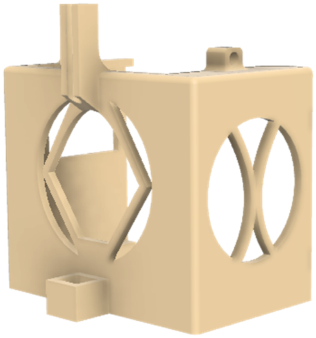
Height-blocker	1 piece, optional.<br><br>Is used to imitate table higher than it really is. Helps to avoid needle crushing by moving up table.	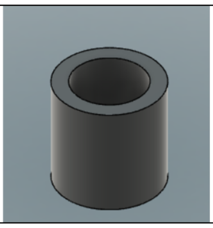
Syringe	1+,<br>The more viscous liquid is used, the more stiff syringe has to be used.	

Tube pipeline	1+,<br>Different diameters and materials are possible. We recommend PPTF tube, inner diameter is 0.3 -0.5 mm, outer diameter is 3 mm.	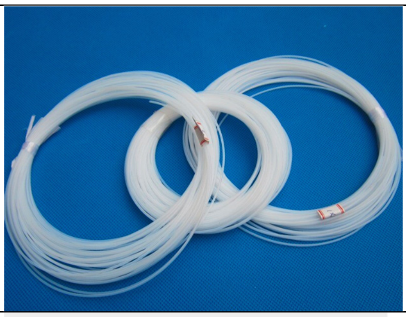
Needle g14-g25 long	5+ of each.<br>We recommend to get reserve needles in advance.	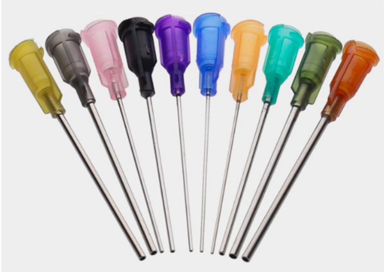
Needle g32 metal	5+<br>The thinner needle you use, the smaller print is possible.	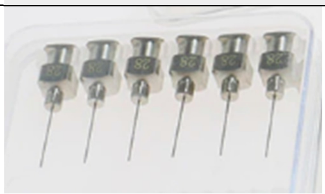

Plunger

Main body	1 piece.<br>Part that holds most parts of plunger together.	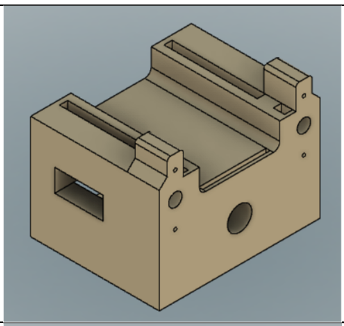
Motor holder	1 piece.<br>House for stepper motor. Together with main body and guide rails form stiff frame of plunger.	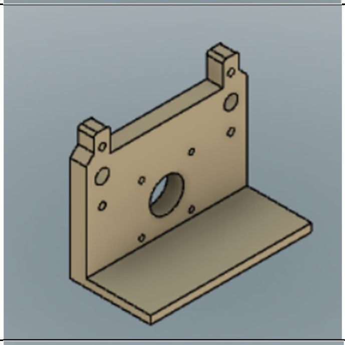
Pusher	1 piece.<br>Pusher of syringe, has grooves for guide rail bearing and lead screw bearing	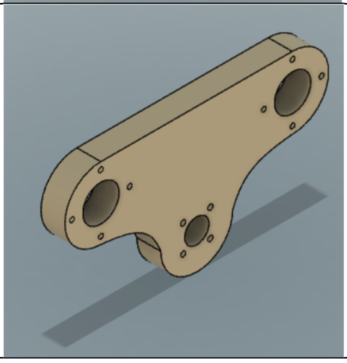

Cylindrical guide rail	2 pieces.<br>150x8 mm.	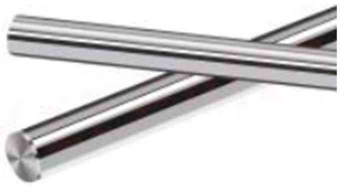
Guide rail bearing for pusher	2 pieces.<br>Inner diameter 8 mm, outer diameter 15 mm. Different outer diameter is possible, but Pusher has to be corrected. Free type.	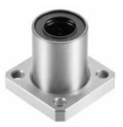 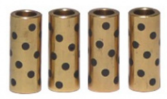
Guide rail holder	For 8mm cylindrical guide rail, 4 pieces.	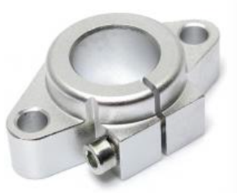
Lead screw + nut	1 pair.<br>d = 8 mm, lead = 2 mm, one threaded.	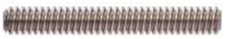 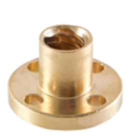
Bearing for lead screw	1 piece, not necessary.<br>Inner diameter 8 mm, outer diameter 15 mm. Different outer diameter is possible, but Main Body has to be corrected.	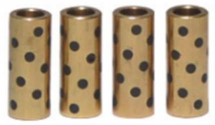
Coupler	1 piece.<br>6 mm to 8 mm, clearance-free.	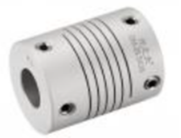
a. Screws<br>b. Self-tapping screw<br>c. Nuts	a. 4 pieces, d=3mm, l = 30 mm<br>b. up to 12 pieces, d=3 mm, l = 10-15 mm.<br>b. up to 12 pieces, d=3-4 mm, l = 20 mm.<br>c. 8 pieces, i.d. = 3 mm	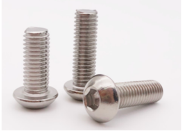 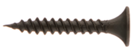 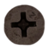 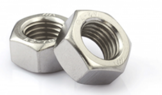
Syringe holder	1+ pieces.<br>Printed on demand on stock printer with changed curvature for syringe of different type.	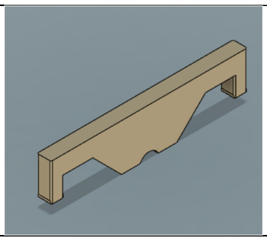

<table>
<tr><td>Nema 17 stepper motor</td><td>1 piece.</td><td>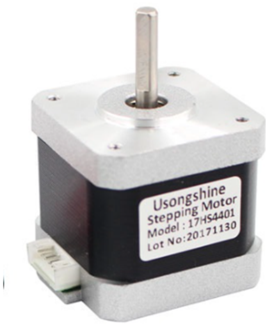
</td></tr>
</table>

II. **Drop code structure.**

The first code for droplets printing consists of two files: file of user interface and calculation file, figure 3. The former is a place where parameters of the printing should be entered, the latter contains functions and classes. Thus, there are two classes and several functions. The first class is Coordinates, that take user input from UI file and returns list of coordinates. This class possesses several functions to produce coordinates either for droplets of for layers. In case of new specific pattern to print one has to create a new function in this class. Once calculations are done, the list is transferred to GCodeInstructions class. There coordinates and user instructions are merged in demanded way, and the result is list of g-code instructions for printer. That class is responsible for changing of initial positions or printing conditions. To achieve new printer behavior while printing that class has to be updated.

The pumping process is split to three different processes: moving toward a point, pumping a droplet, touch-&-back move. This structure provides the highest precision of the print. While an attempt to join this steps into one do leads to faster print, it also results in changed position or size of printed drops.

Finally, function write_in_file just write line by line the list of instruction. G-code instructions file is transferred to printer via Pronterface. That software is comfortable to monitor the printing process.

III. Linear printing code

A primitive script that takes ‘file_name.gcode’ file that is created by Cura-slicer and creates new ‘file_name_first.gcode’ file with instructions, suitable for gel printing. In particular, parameters to enter:

- ‘coordinates_init’ states the [x, y, z] position in the ‘file_name.gcode’ file, created by Cura
- ‘coordinates_desire’ states the [x, y, z] position in a planning print
- ‘PUMP_CONST = 0.120’ the constant that is used to change the length of the printed plastic (that is used by Cura) to the length of the syringe piston propulsion. In general, it is just a coefficient that you experimentally find for particular needle and particular thickness of print. A higher value results in more intensive gel pumping. The value 0.120 we find to be suitable for needle g25 and 0.2 thickness of gel layer at 500 mcl syringe.
- Path to files (foo_bar is nothing but just a name):
  - init_file = r'C:\blablabla\blabla\bla\foo_bar.gcode
  - final_file = r'C:\blablabla\blabla\bla\another_foo_bar.gcode'

When run, script outputs the also length of piston propulsion. Be sure to check you have enough gel in syringe to print.

IV. a.Preparation of printer.

0. Switch on laptop, printer, start Pronterface.
1. Remove all headers. Empty printing space and stock printhead is needed.
2. Calibrate printer by pressing House bottom

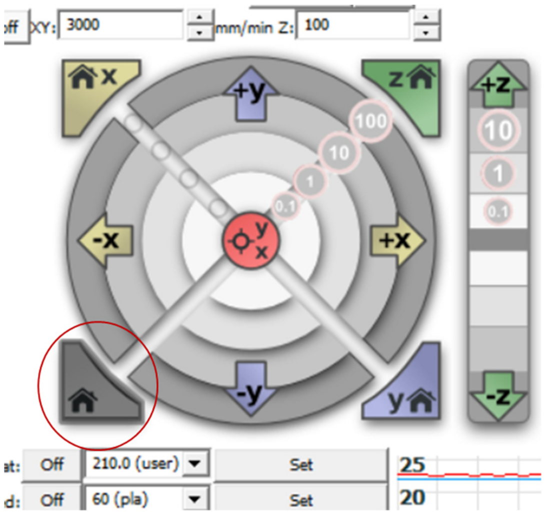

3. Double click '+Z10 mm' to lower the print table; center the printhead

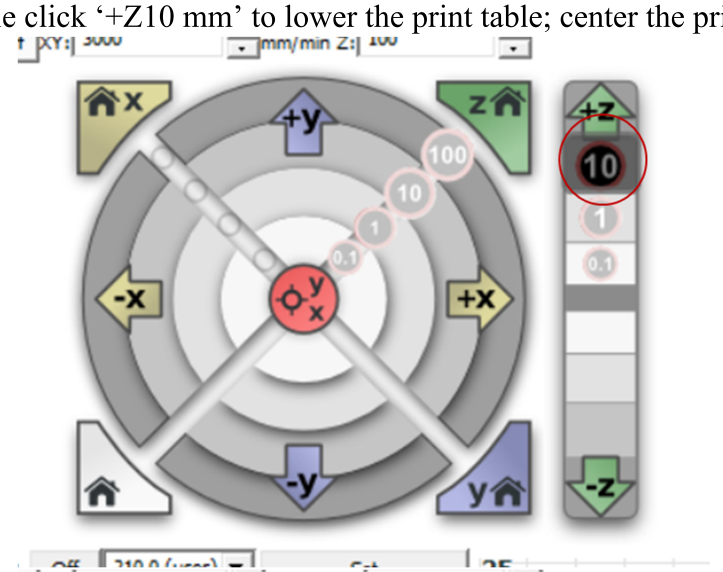

a.

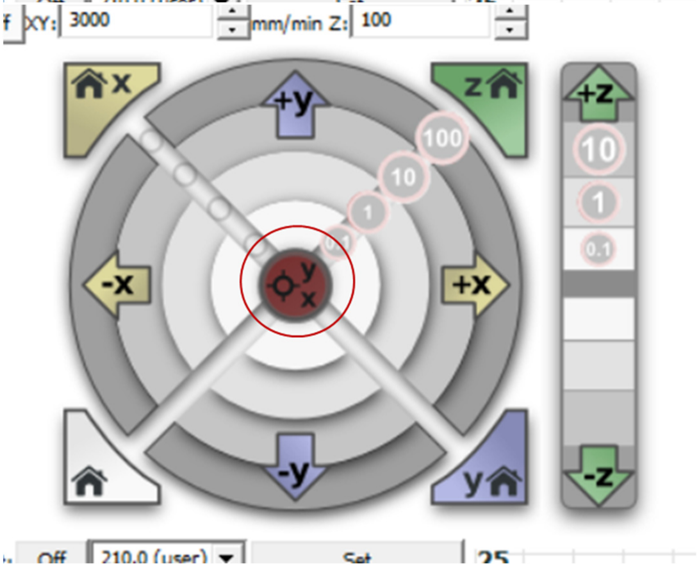

b.

4. Put the header, connect pump line, check the connection of plunger.
5. In Pronterface, enter commands one by one:
    a. M302 S0 – cancel cold extrusion protection
    b. M92 E160 – 10 au of E coordinate set to be equal to π, i.e. half a circle.
    c. G92 E0 – set E-coordinate of piston to 0
    d. M107 – switch of sample cooling fan
6. Set the height-holder, if needed.

IV. b. Drop print methodic

1. Put the substrate on the printing table. Surface has to be horizontal. If the substrate is less, then 20x20x1 mm, we recommend to fix it since it could glued to needle with inks. Use adhesive gum, for example.
2. Move needle to the beginning of the print.
    a. **Slowly** up the table, using **–Z10**, **-Z1** up to ~1 mm height between the needle and the substrate.
    b. Move needle to the start in XY plane using corresponding bottoms ±X(0.1, 1, 10), ±Y(0.1, 1, 10).
    c. **Slowly** up the table using **-Z0.1** up to the needle touching the substrate. Once have touched, make one 0.1 step back.
3. Create instruction file.
    a. Enter command M114. The return is precise coordinates of the needle.
    b. Open script Mapping in g-code, User Interface
    c. Change coordinates of starting point and height
    d. Set drop size, change pattern, and other, if needed.
    e. Name the file
    f. Run the script
    g. Check "Done!", otherwise debug.
4. If the needle was more, then 5 min in air without pumping, one should pump inks though to remove dried layer.
    a. Lower the table by 15 mm and move needle to side 5 cm.
    b. Put water/solvent under the needle and soak the needle.
    c. Pump the gel: enter G1 F10 E5
    d. Up needle from water and continue pumping: G1 F10 E10
    e. Remove formed droplep
    f. Enter G92 E0
5. Start printing:
    a. Load file with instructions

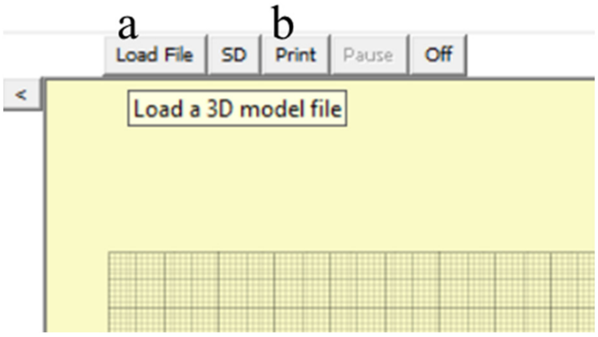

    b. Press Print
6. After printing: lower the table, remove sample. To continue printing, repeat from 1. If ended, we recommend store needle in solvent to prevent clogged end.

IV. c. Continuous print methodic

1. Put the substrate on the printing table. Surface has to be horizontal. If the substrate is less, then 20x20x1 mm, we recommend to fix it since it could glued to needle with inks. Use adhesive gum, for example.
2. Move needle to the beginning of the print.
    a. **Slowly** up the table, using **–Z10**, **-Z1** up to ~1 mm height between the needle and the substrate.

b. Move needle to the start in XY plane using corresponding bottoms ±X(0.1, 1, 10), ±Y(0.1, 1, 10).
c. **Slowly** up the table using **-Z0.1** up to the needle touching the substrate. Once have touched, make one 0.1 step back.
d. Enter M114 and remember the output for step 4.

3. Open Cura project. Load .stl file. Choose type and density of infill as you wish.

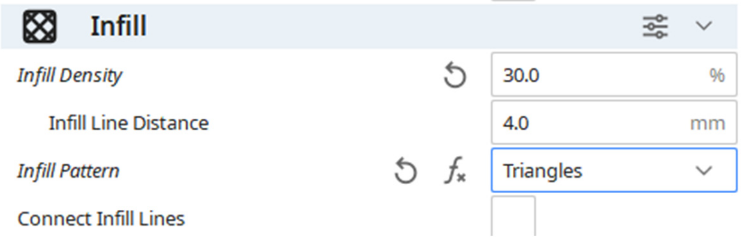

Slice – 3D object to .gcode instructions.

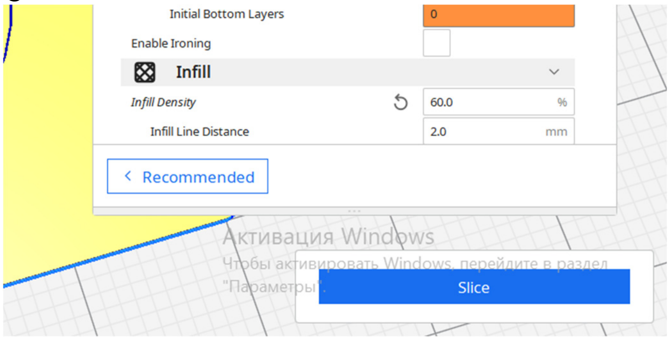

You can check planning printing with Preview.

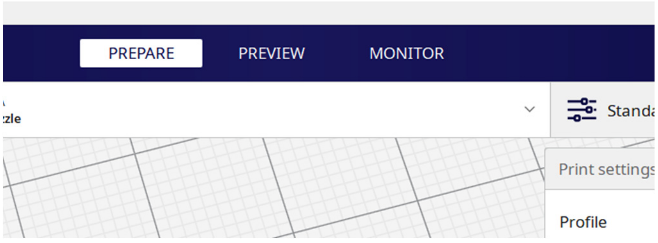

Save to Disk.

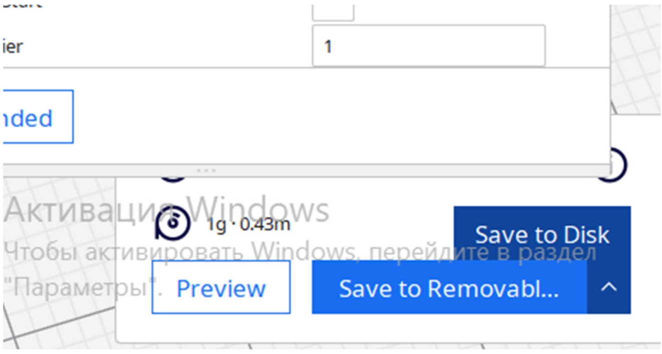

4. Open script “rewrite file”. Set “init_file” as path to .gcode file created earlier in 3. Set a new name in “final_file”, including folder that will contain it. The name has to be like “name.gcode”.

```
# Path to file is C;\blablabla\blabla\bla\foo_bar.gcode
# foo_bar is nothing but just a name
init_file = r'C:\Users\senja\OneDrive\Документы\Work\Labor
final_file = r'C:\Users\senja\OneDrive\Документы\Work\Labo
```

In file from 3) find the coordinates of start (first coordinates after “layer:0” and place them as coordinates_init.

You can you Notepad to open it. Cntrl + F: ;layer:0

```
G92 E0
G92 E0
;LAYER_COUNT:25
;LAYER:0
M107
;MESH:Surprising Inari-Habbi.stl
G0 F6000 X100.2 Y119.8 Z0.2
```

The last line on the picture is starting coordinates.

The real starting position that you got in step 2 has to be entered as “coordinates_desire”.

```
coordinates_init = [98.44, 98.467, 0.2]
coordinates_desire = [100, 130, 11.7]
```

5. Run script. The result is .gcode instructions for Pronterface.
6. Start printing:
    a. Load file from step 4-5 to

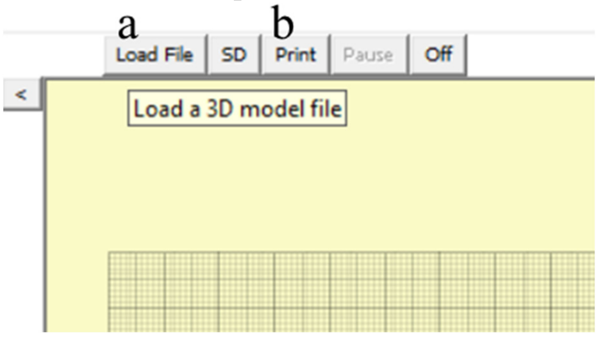

    b. Press Print
7. To continue printing, repeat from 2. If ended, lower the table and remover the sample.

V. List of files enclosed

File	Comment
Appendix 1. Drop printing python scripts	Python scripts for creation of droplet
Appendix 2. Continuous printing python script.	Python scripts to rewrite gcode instructions for continuous printing modes
Appendix 3. Git-hub link	The whole project in internet source to download. LINK
Appendix 4. Cura project	Saved parameters for linear printing in Cura slicer. This file is not necessary, but recommended in case you are new in 3D printing
Appendix 5. Squre.stl	3D model of square with thickness of 2 mm. It is used to be imported in Cura for further slicing.
Appendix 6. Squre.gcode	Previous file sliced. Implied to be feeded to python script. Also suitable for classical 3D printing.
Appendix 7. Squre_first_slice.gcode	The g-code instructions after python script is done. Suitable for particular printer with particular sample in particular location.
Appendix 8. Stl files for plunger and printer	All files that has to be printed on stock FDM 3D printer in order to re-equip it with them. Zip archive.
Appendix 9. Video	File 1, file 2, file 3

VI. Properties and appearance of 3D printed structure.

By using alginate inks we have printed 7 layer structure to show the ability of creation of 3D objects (Fig. S1A).

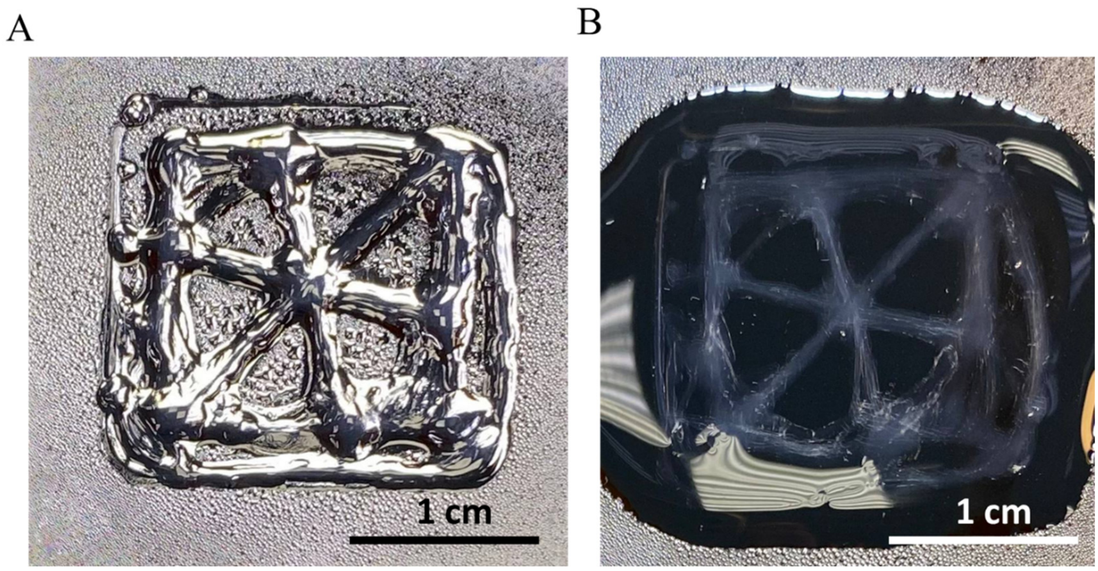

Figure S1. 7-layer printed structure of alginate inks (A) after printing and after immersing in saturated $CaCl_2$ solution (B).

To fixate the shape of printed figure it was immersed in saturated $CaCl_2$ solution (Fig. S1B). After reaction of carboxilic group of inks with calciym ions, all inks shown increase in mechanical properties (Fig. S2A) with preserving main structure (Fig.S2B).

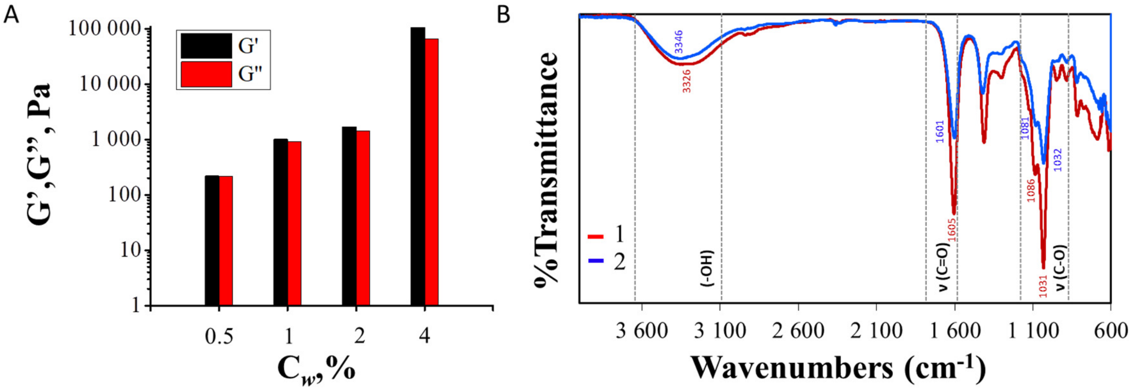

Figure S2. A - modules of accumulation (G′) and loss (G″) of a sample containing 0.5, 1, 2 and 4 wt.% sodium alginate treated with calcium chloride; B - FTIR spectra: 1- sodium alginate, 2 - sodium alginate after treatment with calcium chloride
